# Survey on human milk feeding and enteral feeding practices for very-low-birth-weight infants in NICUs in China Neonatal Network

**DOI:** 10.1186/s12887-023-03862-0

**Published:** 2023-02-11

**Authors:** Xiaoshan Hu, Junjie Lu, Jun Zhang, Min Zhang, Zhangbin Yu, Shoo K. Lee, Shuping Han, Xiaohui Chen

**Affiliations:** 1 Department of Pediatrics, Maternity Hospital Affiliated to Nanjing Medical University/ Nanjing Maternal and Child Health Hospital, Nanjing, Jiangsu China; 2 Department of Pediatrics, University of Toronto Faculty of Medicine, Toronto, ON Canada

**Keywords:** Human milk feeding, Enteral feeding practice, Neonatal intensive care units, Survey

## Abstract

**Background:**

The breastfeeding rate in China is lower than that in many other countries and the extent of adoption of the “Feeding Recommendations for Preterm Infants and Low Birth Weight Infants” guideline in NICUs remains unclear.

**Method:**

A web-based survey about the current status of human milk feeding and enteral feeding practices at NICUs was sent to all China Neonatal Network’s cooperation units on September 7, 2021, and the respondents were given a month to send their responses.

**Results:**

All sixty NICUs responded to the survey, the reply rate was 100%. All units encouraged breastfeeding and provided regular breastfeeding education. Thirty-six units (60.0%) had a dedicated breastfeeding/pumping room, 55 (91.7%) provided kangaroo care, 20 (33.3%) had family rooms, and 33 (55.0%) routinely provided family integrated care. Twenty hospitals (33.3%) had their own human milk banks, and only 13 (21.7%) used donor human milk. Eight units (13.3%) did not have written standard nutrition management guidelines for infants with body weight < 1500 g. Most units initiated minimal enteral nutrition with mother’s milk for infants with birth weight ˂1500 g within 24 h after birth. Fifty NICUs (83.3%) increased the volume of enteral feeding at 10–20 ml/kg daily. Thirty-one NICUs (51.7%) assessed gastric residual content before every feeding session. Forty-one NICUs (68.3%) did not change the course of enteral nutrition management during drug treatment for patent ductus arteriosus, and 29 NICUs (48.3%) instated NPO for 1 or 2 feeds during blood transfusion.

**Conclusion:**

There were significant differences in human milk feeding and enteral feeding strategies between the NICUs in CHNN, but also similarities. The data obtained would be useful in the establishment of national enteral feeding guidelines for preterm infants and quality improvement of cooperation at the national level.

**Supplementary Information:**

The online version contains supplementary material available at 10.1186/s12887-023-03862-0.

## Background

Human milk (HM) is the most ideal source of nutrition for newborns, especially preterm infants, on account of its short-term benefits, such as reducing the risk of necrotizing enterocolitis (NEC), sepsis, chronic lung disease(CLD), and mortality, and its long-term benefits, such as improving long-term neurodevelopment [1]. Apart from its medical advantages, HM feeding also has economic advantages [2]. Lactation onset is often delayed after premature delivery, and as a result, preterm infants receive an insufficient amount of milk during the first few critical days of life [3]. Donor human milk (DHM) and preterm formula are used as supplements when MOM is insufficient, and DHM is preferred over preterm formula as it is better in terms of nutritional composition and biological value [4, 5].

Therefore, the World Health Organization and the American Society for Parenteral Nutrition have emphasized the use of HM in preterm infants in their guidelines for the nutritional management of preterm/low-birth-weight infants [6]. Similarly, China issued “Feeding Recommendations for Preterm Infants and Low Birth Weight Infants” in 2009, according to which mother’s own milk (MOM) was recommended as the first choice for preterm infants [7]. Following these guidelines, the breastfeeding rate in China’s NICUs increased from 23% in 2009 to 58.2% in 2018 [8]. However, the breastfeeding rate is still far lower than that in other countries [9]. Furthermore, the situation of implementation of “Feeding Recommendations for Preterm Infants and Low Birth Weight Infants” for each NICU was still unclear. Therefore, the purpose of this study was to examine and compare the status of HM feeding and enteral feeding strategies in China’s level 3 NICUs. This is the first national-level survey on this topic in China, so the data obtained would be useful in the establishment of national enteral feeding guidelines for preterm infants and quality improvement of cooperation at the national level.

## Methods

### Survey methods

On September 7, 2021, a prospective, cross-sectional, web-based survey was sent to the director or representative of 60 level 3 NICUs participating in the Chinese Neonatal Network (CHNN).The CHNN is the first national neonatal network and has the largest geographically representative cohort from NICUs in China. It was officially put into operation on January 1, 2019. CHNN hospitals are tertiary referral hospitals with Grade A level 3 NICUs authorized by the Health Administration of China and have recognized expertise in caring for high-risk neonates.

The survey contained questions about the current status of HM feeding and enteral feeding strategies,and the unit director or representative was responsible for completing the survey and was instructed to provide answers based on common unit-level practice rather than their personal opinions/practice alone. The questionnaire, which consisted of single-choice, multiple-choice, and open-ended questions, gathered information about the demographic features of the unit, HM feeding characteristics, presence of a milk bank or access to DHM, initiation and advancement of enteral feeding, and indications for and the use of human milk fortifier (HMF), among other data(see supplementary materials). The deadline for submitting the filled-in surveys was October 7, 2021.

### Data collection and analysis

Participants were enrolled between September 7 and October 7,2021. A link to the online questionnaire was sent through WeChat. Participants only needed to fill in the basic information for the NICUs and no personal information was attached to the data. All questions had to be completed before submission, duplicate responses were verified and eliminated.

The survey results were collected online and all analyses were conducted using Microsoft Excel. The demographic information was described by median (interquartile range,1–3). Descriptive results are expressed as numbers and percentage (%).

## Results

Sixty NICUs responded to the survey: 14 (23.3%) were from children’s hospitals; 23 (38.3%) maternity hospitals; and 23 (38.3%) general hospitals. The median number of beds in these units was 100, and the median number of neonates hospitalized in 2020 was 3000, including 907 premature infants, 140 very-low-birth weight (VLBW) infants, and 148 infants of gestational age < 32 weeks (Table 1).

**Table 1:** Demographic information

	Number of total beds in 2020	Number of neonates hospitalized in 2020	Number of premature infants hospitalized in 2020	Number of VLBW infants hospitalized in 2020	Number of GA < 32w infants hospitalized in 2020
*M* (IQR,*1–3*)	100(63–150)	3000(1995–4153)	907(590–1505)	140 (89–251)	148 (87–273)

*M* means median, *IQR* means interquartile range, *IQR1* means the 25th percentile and *IQR3* means the 75th percentile (*N* = 60); *VLBW* very low birth weight; *GA* gestational age

### Education about breastfeeding

All 60 units encouraged breastfeeding at their NICUs. Parents were educated about breastfeeding before delivery at 26 units (43%), at the time of neonate admission at 57 units (95%), or during hospitalization at 57 units (95%). The staff involved in breastfeeding education included neonatal physicians at 51 units (85%), obstetricians at 21 units (36%), nurses in neonatal units at 60 units (100%), and nurses at human milk banks (HMBs) at 17 units (28%). The content and mode of communication of breastfeeding education for parents are shown in Table 2. Most of the units would repeat the breastfeeding instructions to the parents before neonatal discharge from the hospital (Table 2).

**Table 2:** Education about breastfeeding

Content	NO	Mode	NO	Repeat breastfeeding instructions before discharge	NO
benefits of breastfeeding	60	oral instruction	59	importance of breastfeeding	56
collection,storage and transport of breast milk	60	Video	37	how to maintain lactation	50
kangaroo care	52	paper materials	57	feeding advises after discharge	58
how to maintain lactation	59	bedside education	39	the phone number of Breastfeeding counselling clinic	44
Feedback of breastfeeding	52	WeChat or SMS platform	40	Fellow-up after discharge	59

### Feeding with mother’ own milk

Out of the 60 units, 36 (60.0%) had a dedicated breastfeeding/pumping room for mothers. Only 2 units (3.3%) did not accept MOM during neonatal hospitalization, and 36 units (61.7%) did not routinely pasteurize MOM after collection. Further, 55 units (91.7%) provided kangaroo care (KC), 20 units (33.3%) had family rooms, and 33 units (55.0%) routinely carried out family integrated care (FICare, parents spent ≥ 6 h per day in the NICU and provided non-medical care for their infants under the close supervision of the FICare nurse).

### Donor human milk and human milk bank

Twenty of the hospitals (33.3%) established their own HMB, and the sources of operating funds for the HMB were social funding at 4 hospitals (20%), hospital funding at 15 (75%), and own unit funding at 14 (70%). All the HMBs provided their services for free, for both donors and recipients of DHM.

Only 13 units (21.7%) were licensed to use DHM. The main reasons for using DHM were to reduce the risk of NEC and support mother’s breastfeeding. The most common reason for not using DHM was the lack of a license. DHM was most frequently used in cases of extremely/very low birth weight infants, feeding intolerance, and intestinal malabsorption (Table 3).

**Table 3:** Donor human milk and human milk bank

Reasons for providing donor human milk	NO	Reasons for not providing donor human milk	NO	Situations in which donor milk is used	NO
reduce NEC	13	Expensive	7	ELBW/VLBW	13
Improve feeding tolerance	11	Patents not receptive	14	Intestinal malabsorption	7
Reduce allergies	9	Milk bank guidelines inadequate	20	feeding intolerance	12
Family request	1	No beneficial	0	immunologic deficiency disease	0
Support mother’ breastfeeding	12	Inadequate growth	1	Malnutrition after surgery	5
Provide exclusively human milk feeding	8	Reduce mother’ milk production	1	Severe infection	1
Reduce nosocomial infection	7	No license	38		

### Enteral feeding strategies

**Fig. 1:**
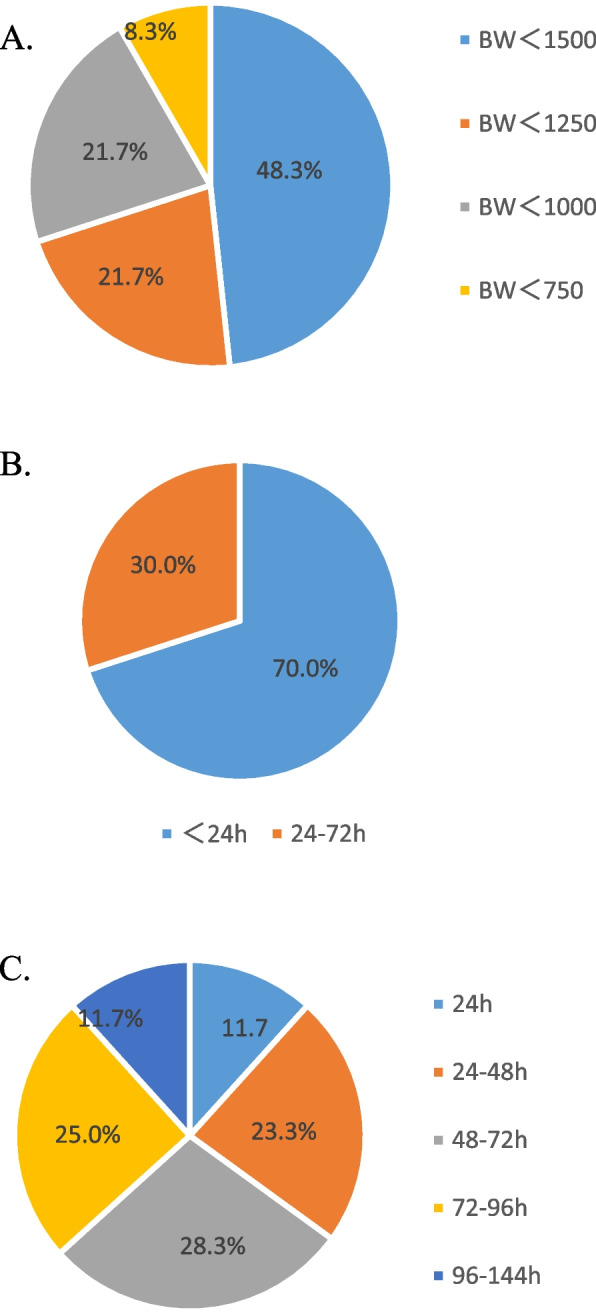
Minimum enteral nutrition practices at NICUs. **A** Infants that require minimal enteral nutrition after birth; **B** Time at which minimal enteral nutrition was started after birth for infants with body weight < 1500 g; **C** Duration of minimal enteral nutrition for premature infants

Eight units (13.3%) did not have any standardized protocol for nutrition management for infants with body weight < 1500 g. Twenty-nine units (48.3%) initiated minimal enteral nutrition (MEN,involves feeding with low volumes of 12–24 ml/kg/day in the first week of life) for infants with birth weight < 1500 g within 24 h after birth, and used fresh mother’ milk. However, 13 units (21.7%) used < 1250 g body weight as the criterion for initiating MEN, 13 units (21.7%) used < 1000 g and 5 units (8.3%) used < 750 g (Fig. 1A and B). The duration of MEN ranged from 24 to 144 h (Fig. 1C).

**Fig. 2:**
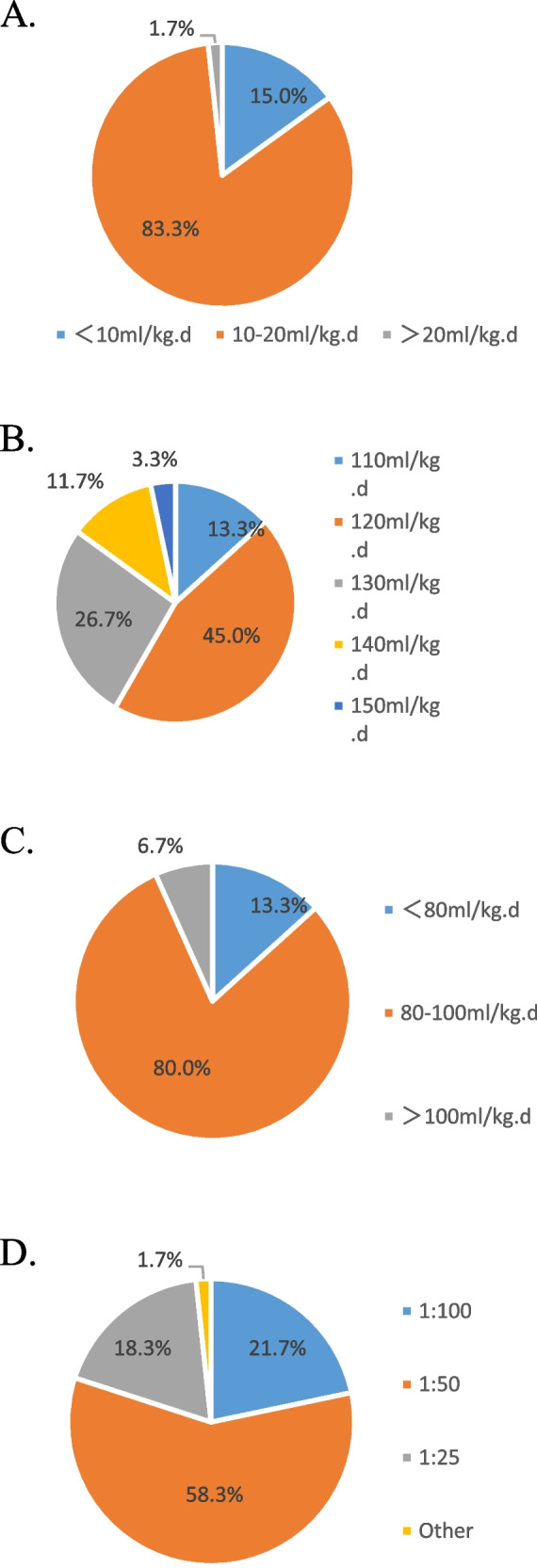
Enteral feeding and human milk fortifier at NICUs. **A** Rate at which the volume of enteral nutrition was increased in premature infants; (**B**) Volume of enteral nutrition required to discontinue parenteral nutrition; (**C**) Volume of enteral nutrition before the use of human milk fortifier was initiated; (**D**) Ratio of human milk fortifier at the time of initiation

Most of the NICUs increased the volume of enteral feeding at the rate of 10–20 ml/kg daily (Fig. 2A) and discontinued parenteral nutrition when the enteral volume reached ≥ 120 ml/kg daily (Fig. 2B). HMF was added when enteral volume reached 80–100 ml/kg daily in most units(80%), at the ratio of 1:50 (HMF to human milk) as initial dosage(58.3%) (Fig. 2C and D).

**Fig. 3:**
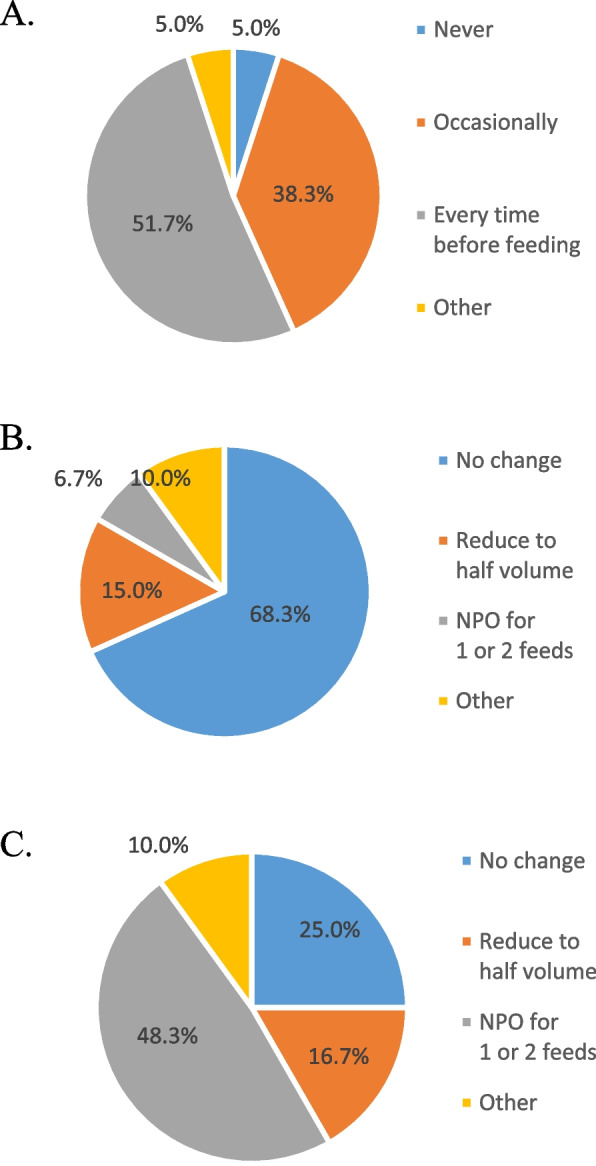
Gastric residual content and changes in feeding strategy. **A** Time point for assessment of gastric residual content; **B** Changes in enteral feeding strategies during drug treatment for patent ductus arteriosus; **C** Enteral feeding changes during blood transfusion

At 31 NICUs (51.7%), gastric residual content was examined before every feeding episode (Fig. 3A). At 41 NICUs (68.3%), the course of enteral nutrition management was not changed during treatment for patent ductus arteriosus(PDA)(Fig. 3B). At 29 NICUs (48.3%), enteral feeding was stopped for 1 or 2 feeds during blood transfusion to prevent NEC (Fig. 3C).

## Discussion

Nutrition is important to standardize among different NICUs, even in terms of long-term outcomes. For example, the prevention of extra-uterine growth retardation (EUGR) in preterm infants through nutritional strategies is of extreme importance, as the achievement of adequate growth has been associated with a better neurodevelopmental outcome through childhood [10], although there is still no consensus regarding the best definition of EUGR to use to predict neurological outcome [11].

This is the first comprehensive study to investigate human milk feeding and enteral feeding practices in NICUs from all districts in mainland China. As evident in the survey responses, we found significant variations but also similarities in the status of human milk feeding and enteral feeding strategies among the examined NICUs. In general, all the NICUs encouraged breastfeeding, and breastfeeding education was provided by nurses in the neonatal units. The content of breastfeeding education covered benefits of breastfeeding, collection,storage,and transport of breast milk,kangaroo care, how to keep lactation,feedback of breastfeeding. Breastfeeding information was provided via channels such as oral instruction, video, paper materials,bedside education,WeChat or SMS platform and others. During the recent COVID-19 pandemic, parental visitation practices were suspended at many NICUs in China but once the pandemic was brought under control, most NICUs restarted their previous parental visitation policies. At most NICUs, communication about breastfeeding with parents was implemented online or through face-to-face meetings at least once a week. Further, at the majority of the hospitals (58 NICUs, 96.7%), MOM was obtained during neonatal hospitalization.

The present findings showed significant variations between the NICUs with regard to the provision of family rooms and FICare, the use of DHM, and enteral feeding strategies.55 NICUs (91.7%) provided KC. Accordingly, previous studies have documented that most NICUs in China were aware of the benefits of KC [12, 13]. Compared to the high rates of implementation of KC, family rooms were provided in only 20 NICUs (33.3%) and only 33 units (55.0%) routinely provided FICare. Family rooms are known to be beneficial for both infants and parents, as they improve weight gain, promote breastfeeding after NICU discharge, reduce parental stress and anxiety, as well as reduce the incidence of health-assistance related infections [14]. Additionally, FICare in Chinese NICUs was found to be associated with reduced hospital length of stay, medical expenditures, and rates of adverse outcomes [15]. Therefore, in the future, NICUs in China should promote FICare vigorously.

Since the first HMB was established in Guangzhou in 2003, 20 HMBs have been established in China so far. All these HMBs provide their services free of charge to both donors and recipients of DHM. However, the cost of running HMBs is quite high [16] and is almost entirely borne by the local hospitals, as shown by our findings. This is also one of the reasons for the small number of HMBs in China. Therefore, it is necessary to find alternate sources of funds for HMBs in China. In China, both parents and medical workers’ attitude about DHM is positive [17–19], but in our survey, only 13 units (21.7%) used DHM. The most common reason for not using DHM was the lack of a license. This highlights the need to improve the process of using DHM in China.In the 7 units that established an HMB but did not use DHM, the reason was because the banks were awaiting for their licensing.Therefore, they could only manage and use MOM in the HMB.

Our study highlights the diversity of recommended feeding practices in NICUs across China. While most of the units initiated MEN for infants with birth weight < 1500 g (48.3%), the criteria for initiating MEN varied considerably across the remaining units. The optimal duration of MEN is still under debate [20, 21], and this was also reflected in the results of our study. With regard to feed volume, we found that most units (83.3%) increased enteral feeding intake by 10 to 20 ml/kg daily. A Cochrane review compared daily feed intake increments of 15 to 20 ml/kg versus 30 to 35 ml/kg and concluded that more rapid advancement did not increase the risk of NEC, mortality, or interruption of feeds [22]. Further, previous studies reported that early fortification (< 40 ml/kg daily) was safe and well tolerated [23]. In the present study, we found a significant variation in both the recommended timing of initiation and the recommended initial volume of HMF. Another international survey-based study also reported large variations in the timing of initiation and volume of HMF at initiation [24]. Given these variations in enteral feeding strategies and the previous findings, national-level guidelines should recommend standardized enteral feeding and human milk fortification strategies for NICUs in China.

Routine assessment of gastric residual content was reported by 31 units (51.7%).A previous study indicated that the omission of gastric residual evaluation increased the delivery of enteral nutrition as well as improved weight gain and led to earlier hospital discharge [25]. This suggests that the NICUs in China should probably change the previous practice.A previous study reported that feeding during pharmacological PDA closure in preterm neonates was not associated with delay to reach full feeds, NEC incidence, or gastric residuals; nonetheless, feeding management of this population should be carefully evaluated [26]. Our study showed that 41 units (68.3%) did not make any changes in their enteral feeding regimen during drug treatment for PDA. Further, feeding during blood transfusions may increase the risk for mesenteric ischemia and the development of transfusion-related NEC in preterm infants [27]. In the present study, 29 units (48.3%) instated stop feeding for 1 or 2 feeds during blood transfusion, but 15 units (25.0%) reported that did not introduce any changes and only closely monitored the situation.

There are some limitations to this study. As with other surveys, a responder bias could not be ruled out, and the responses may, potentially, not be representative of the practices of the unit. We also did not evaluate whether the variation in feeding practices are associated with infant growth and outcomes such as NEC.

## Conclusion

There were significant differences in human milk feeding and enteral feeding strategies between the NICUs in CHNN, but also similarities. Establishing national-level feeding guidelines for preterm and low birth weight infants and quality improvement of cooperation at the national level are needed. This is the first national-level survey on this topic in China, so the data obtained would be useful in the establishment of national enteral feeding guidelines for preterm infants and quality improvement of cooperation at the national level.

## Supplementary Information

**Additional file 1.**

## Data Availability

The datasets used and analysed during the current study are available from the corresponding author upon request.
