# Supplementary material for: Survey on human milk feeding and enteral feeding practices for very-low-birth-weight infants in NICUs in China Neonatal Network
Source: BMC Pediatr. 2023 Feb 11;23:75. doi: 10.1186/s12887-023-03862-0 (PMC9918398; doi:10.1186/s12887-023-03862-0)
Supplement: Supplementary file 1 — Additional file 1. [file 12887_2023_3862_MOESM1_ESM.docx]

**Survey of Human Milk Bank and Human Milk Feeding in China**

**Part 1 -****Hospital Information**

1. Name of hospital:______
2. Type of hospital:
3. Children’s hospital (2) Maternal and Infant Hospital (3) General Hospital
4. Number of beds in your unit:______
5. Number of newborns hospitalized in your unit in 2020:______
6. Number of premature infants hospitalized in 2020: ______
7. Number of premature infants with low birth weight (<1500g) hospitalized in 2020:______
8. Number of premature infants with GA <32w hospitalized in 2020: ______

**Part 2- Breastfeeding Education- in direct rooming-in**

1. Does your hospital accept and encourage breastfeeding?

(1)Yes (2) No

1. Does your delivery room routinely carry out skin-to-skin care and early newborn suckling?

(1)Yes (2) No

1. When will the parents be educated about breastfeeding in your hospital? (multi-choice)

(1)Never (2) Before delivery (3) On admission (4) During postnatal hospitalization (5) Before discharge

1. The staff for breastfeeding education includes: (multi-choice)

(1)Obstetricians (2)Maternity nurse (3) Certified Lactation Consultant Specialists

1. The breastfeeding education above-mentioned includes: (multi-choice)

(1)Benefits of breastfeeding (2) Breast nursing posture and position (3) Key point of latching (4) How to maintain lactation (5)Handling of common issues with breastfeeding (6) Storage of breast milk

1. The channels for the spreading breastfeeding education above-mentioned include: (multi-choice)

(1)Oral instruction (2) Video (3) Paper materials (4) Bedside education (5) WeChat or SMS platform (6) Others:______

1. Do the nurses regularly evaluate the situation of breastfeeding during postnatal hospitalization? What is the frequency?

(1)Once a day (2) Three times a day (3) more than three times a day

1. How do nurses evaluate breastfeeding?

(1) Changes in the maternal breast (2)Newborn defecation (3)Newborn body weight (4) Do not evaluate.

1. Do the puerperant get repeated breastfeeding instruction before discharge from hospital? The education includes : (multi-choice)

(1)No re-education（2）Importance of breastfeeding (3) How to maintain lactation (4)Information on the phone number of Breastfeeding Counselling Clinic (5) Follow-up options after discharge

**Part 3- Breastfeeding Education-in NICU**

1. Does your NICU accept and encourage breastfeeding?
2. Yes (2) No
3. When will the parents be educated about breastfeeding in your hospital? (multi-choice)
4. Never (2) Before delivery (3) On admission (4) During neonatal hospitalization (5) Before discharge
5. The staff for breastfeeding education includes: (multi-choice)
6. Obstetricians (2) Neonatal physicians (3) Maternity nurse (4) Neonatal nurses (5) Breast-milk Bank Nurse (6) Certified Lactation Consultant Specialists
7. The breastfeeding education for parents separated from their preterm infant includes: (multi-choice)
8. Benefits of breastfeeding (2) Collection, Storage and Transport of breast milk (3) Kangaroo care (4) How to maintain lactation
9. The channels for the spreading breastfeeding education during neonatal hospitalization include: (multi-choice)
10. Oral instruction (2) Video (3) Paper materials (4) Bedside education (5) WeChat or SMS platform (6) Others:______
11. Do the doctors or nurses regularly communicate with parents on breastfeeding during neonatal hospitalization? What is the frequency?
12. Once a day (2) Once a week (3) more than once a week but less than once per day (4) Time uncertain (5) No communication
13. Does your unit record the amount of breast milk used in every feed?
14. Yes (2) No
15. Do the mothers in your hospital use lactation logs during parent-infant separations?
16. Yes (2) No
17. Do the parents get repeated breastfeeding instruction before neonatal discharge from hospital? The education includes : (multi-choice)
18. No re-education（2）Importance of breastfeeding (3) How to maintain lactation（4）Duration of breastfeeding after discharge (5) Information on the phone number of Breastfeeding Counselling Clinic (6) Follow-up options after discharge

**Part 5 - Donor human milk and human milk bank**

1. Does your unit have a human milk bank?
2. Yes (2) No
3. Does your hospital use donor human milk?
4. Yes (2) No
5. Reasones for providing donor human milk:
6. Reduce NEC （2）Improve feeding tolarence (3)Reduce allergies （4）Family request (5)Support mother's breastfeeding (6)Provide exclusively human milk (7)Reduce nosocomial infection
7. Reasones for not providing donor human milk:
8. Expense （2）Parents not receptive (3)Milk bank guidelines inadequate （4）Not beneficial (5)Inadequate growth (6)Reduces mother's milk production (7) No license
9. Number of donors of donor breast milk during 2020: ______?
10. Volume of donor milk collected (ml) during 2020: ______?
11. Volume of donor milk used (ml) during 2020: ______ ?
12. Facilities in your human milk bank include: (multi-choice)
13. Hospital-level milk pump (2) Handwashing facilities (3) Human milk storage containers (4) Pasteurizer (Temperature Controlled Water Bath) (5) 2-8℃ medical refrigerator (6) -20℃ medical refrigerator (7) Computer and information management System (8) Human milk composition analysis instrument (9) Super-clean work bench
14. The source of operating funds for the human milk bank: (multi-choice)
15. Social funding (2) Hospital Support (3) Afford by own unit

(4) Profits from human milk bank

1. Does your hospital charge for the usage of donor human milk from the human milk bank?
2. Yes (2) No
3. Staff working in the human milk bank:
4. Doctor (2) Nurse (3) No dedicated staff
5. Do you supply donor milk to other hospitals?
6. Only own hospital (2) Only hospital in our city (3) Only hospital in our province
7. What are the top three common situations for using donor milk in your hospital: (multi-choice)
8. ELBW/VLBW (2) Feeding intolerance (3) Immunologic deficiency disease (4) Malnutrition after surgery (5) Severe infection (6) Others:
9. Are donors screened for: (multi-choice)
10. Smoking (2) Alcohol (3) Drugs (4) Medicines (5) HIV risk
11. Are donors tested for? (multi-choice)
12. Syphilis (2) Tuberculosis (3) Hepatitis B (4) Hepatitis C (5) HIV (6) CMV (7) Other infections
13. Does your human milk bank accept donor milk collected at home?
14. Yes (2) No
15. Does your unit routinely check the macronutrients in breast milk ?
16. Yes (2) No
17. The method of pasteurization for donor human milk:
18. Do not pasteurized (2) 62.5℃ for 30min (3) 75℃ for 15sec
19. Is all donated milk tested bacteriologically before pasteurization?
20. Yes (2) No
21. If no, When do you tested?

(1)On first donation (2) Every donation (3)Occasionally (4)Never (5)Other (please specify)?

1. Is donated milk tested after pasteurization? Always or sometimes?

(1)Always (2) Sometimes (3)Never

1. Can donor milk be tracked from donor to recipient?
2. Yes (2) No

**Part 5 - Mother’s Own milk feeding in NICU**

1. Is there a separated breastfeeding/pumping room in your unit?
2. Yes (2) No
3. What is the permissable time frame for receiving the own mother’s milk in your unit?
4. 24 hours (2) Fixed working time (3) Other:
5. What kind of mother’s milk can be accepted in your unit?
6. Fresh milk (2) Chilled milk (3) Frozen milk
7. What type of container is used for own mother’ milk collection in your unit?
8. Plastic milk storage bottle (2) Glass milk storage bottle (3) milk storage bag
9. Does the information of mother’s milk get recorded in your unit?
10. Yes (2) No
11. The collecting information includes:
12. Collection time (2) Collection volume (3) Acceptance Time (4) Other
13. Is the mother’s milk routinely pasteurized after collection?
14. Not routinely pasteurized (2) 62.5℃ for 30min (3) 75℃ for 15sec
15. Is the mother’s milk routinely tested for CMV?
16. Not routinely tested (2) All tested (3) Tested for BW＜1500g premature infants (4) Tested for BW＜1000g premature infants (5) Tested if suspected CMV infection (6) Other factors (please list)
17. If CMV-DNA of own mother’ milk is positive, do you change the management of breastfeeding?
18. No change (2) freeze the milk (3) pasteurize the milk (4) Stop breastfeeding (5) Other.
19. Does your unit routinely carry out kangaroo care? In which circumstances?
20. Do not routinely carry out (2) For all newborns (3) For all premature infants (4) For GA＜32w premature infants (5) For GA＜28w premature infants （6）Other
21. Does your unit have infant-parent rooms for preterm infants before discharge?
22. Yes (2) No
23. Does your unit routinely carry out family integrated care (FICare) ?
24. Yes (2) No
25. Does your unit permit parents to enter the NICU for visition?
26. Yes (2) No
27. How often are the parents permitted to enter the NICU for visition?
28. Any time (2) Once a day (3) Once a week (4) More than once a week (state frequency per week:____) (5) Less than once a week (state frequency per 2 weeks:____) (6) Other

**Part 7 - Feeding guidelines for BW****<1500g infants**

1. Is there any standard course of nutrition management (written) of BW<1500g infants at your unit?
2. Yes (2) No
3. Who routinely needs minimal enteral nutrition after birth at your unit?
4. Infants with BW<1500g (2) Infants with BW<1250g (3)Infants with BW<1000g (4)Infants with BW<750g
5. When do you start minimal enteral nutrition after birth for BW<1500g infants?
6. Within 24 hours (2) 24-72 hours (3) after 72 hours
7. What are the common reasons for delays in enteral feeding?
8. Asphyxia (2) ELBW/VLBW (3) Invasive ventilation (4) Noninvasive ventilation (5) Use vasopressor (6) Abdominal distension (7) Bloody gastric aspirate (8) Bilious gastric aspirate (9) Waiting for own mother’ milk (10) Other
9. What do you choose for minimal enteral nutrition?
10. Fresh mother’s milk (2) Frozen mother’s milk (3) Donor human milk (4) Premature infant formula (5) Deep hydrolyzed milk
11. Does your unit routinely administer colostrum as oral immunotherapy for BW＜1000g infants?
12. Yes (2) No
13. Duration of minimal enteral nutrition for premature infants?
14. 24 hours (2) 24-48 hours (3) 48-72 hours (4) 72-96 hours (5) 96-144hours
15. At what rate do you increase the volume of enteral nutrition (per day) for premature infants?
16. <10 mL/kg/d （2）10-20 mL/kg/d （3）＞20 mL/kg/d
17. Volume of enteral nutrition before use of human milk fortifier is initiated?
18. ＜80 mL/kg/d （2）80-100 mL/kg/d （3）＞100 mL/kg/d
19. Volume of human milk fortifier at initiation?
20. 1:100 （2）1:50 （3）1:25 (4) other
21. What volume of EN do you generally require in order to discontinue parenteral nutrition?
22. 110mL/kg/d （2）120mL/kg/d（3）130mL/kg/d（4）140mL/kg/d（5）150mL/kg/d
23. Do you routinely check the gastric residual content?
24. Never (2) Sometimes (3) Every time before feeding (4) Other
25. Do you change the course of enteral nutrition management during transfusion?
26. No change (2) Hold the feeds (3) Reduce the volume (4) NPO for 1 or 2 feeds (5) Fasting for 24 hours (6) Others
27. Do you change the course of enteral nutrition management during drug treatment for PDA?
28. No change (2) Hold the feeds (3) Reduce the volume (4) Fasting during treatment (5) Others
29. Does each premature infant get nutritional assessment before discharge in your unit?
30. Never (2) Always (3) Sometimes (please list frequency and rationale)
31. Does each premature infant get personalized breastfeeding guidance before discharge?
32. Never (2) Always (3) Sometimes (please list frequency and rationale)
